# Combined Flexion, Torsion and Compression Drive Distinct Intervertebral Disc Failure Mechanisms Under Asymmetric, High‐Cycle Loading

**DOI:** 10.1002/jsp2.70163

**Published:** 2026-02-11

**Authors:** Amra Šećerović, Aapo Ristaniemi, Francesco Crivelli, Sarah Heub, Mauro Alini, Gilles Weder, Diane Ledroit, Stephen J. Ferguson, Sibylle Grad

**Affiliations:** ^1^ AO Research Institute Davos Davos Switzerland; ^2^ Swiss Center for Electronics and Microtechnology Alpnach Switzerland; ^3^ Swiss Center for Electronics and Microtechnology Neuchatel Switzerland; ^4^ ETH Zürich Institute for Biomechanics Zürich Switzerland

## Abstract

**Background:**

Recent advancements in next‐generation bioreactors have substantially improved the simulation of complex, detrimental spinal mechanics in ex vivo intervertebral disc models. This study investigated intervertebral disc responses to combined flexion, torsion, and static compression. A range of loading frequencies, magnitudes, and patterns was applied to identify conditions that contribute to disc degeneration under complex motion.

**Methods:**

Twelve bovine coccygeal intervertebral discs (mean age 9 months) were subjected to three distinct loading regimes, with four samples per condition. Static compression of 0.1 MPa was combined with: (1) symmetrical 3° flexion/extension and 2° torsion, (2) symmetrical 6° flexion/extension and 4° torsion, and (3) asymmetrical 6° flexion and 4° torsion. Loading frequencies and durations ranged from 0.2 Hz for 1 h in symmetrical loading to 1 Hz for 2 h in asymmetrical loading over a 14‐day period. Structural integrity, cell viability, tissue composition, and molecular responses were evaluated using histology, biochemical assays, and gene expression analysis.

**Results:**

Lower‐cycle symmetrical flexion/extension and torsion, regardless of magnitude, preserved disc structure and maintained a high cell viability (88% ± 14%) across all disc regions. Higher cycle numbers and asymmetrical loading induced significant fissures in the outer annulus fibrosus (AF) on the tensed side (*p* < 0.01) and delamination on the compressed side. This structural damage occurred in AF regions with high cell viability (81% ± 17%), whereas significantly reduced cell viability was observed in the inner AF (30% ± 33%) and nucleus pulposus (28% ± 35%).

**Conclusions:**

Under conditions of asymmetrical and more frequent loading, complex motion involving flexion, torsion, and compression led to structural damage in the outer disc regions and promoted cell death in inner regions. These region‐specific responses suggest the independent development of distinct failure mechanisms contributing to disc degeneration. They also underscore the importance of developing targeted strategies that address both structural integrity and cellular resilience in degeneration models and therapeutic interventions.

## Graphical abstract

Using advanced multiaxial bioreactor systems, this study investigated the effects of flexion, torsion, and static compression on whole intervertebral discs. High cycle asymmetric loading resulted in cell death in the nucleus pulposus and structural changes in the annulus fibrosus. This suggests independent development of distinct failure mechanisms contributing to disc degeneration.

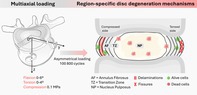

## 1 Introduction

Subjecting ex vivo intervertebral disc (IVD) organ culture models to mechanical loading in bioreactors aims to maintain their homeostasis or induce degeneration [1]. Recent advances in bioreactor technology have significantly improved the ability to replicate complex spinal mechanics, evolving from simple uniaxial displacement to sophisticated six‐degrees‐of‐freedom (6 DOF) loading systems [2, 3]. These developments integrate combined axial displacements and rotational motions, closely mimicking physiological loading conditions, and enabling the investigation of normal disc function and degenerative mechanisms under controlled biological conditions.

The first integration of complex mechanical loading with environmental control was demonstrated using asymmetrical static compression under angular control [4]. Subsequent developments introduced biaxial loading modes involving torsion and bending [5, 6, 7]. More advanced systems capable of simulating 6 DOF motions have since emerged, focusing on biomechanical and structural analyses [8, 9, 10].

Among 6 DOF motions, flexion and torsion are particularly well‐established risk factors for IVD structural changes [11, 12]. Flexion was shown to increase intradiscal pressure [13] and induce maximum shear stresses [14] raising the risk of annulus fibrosus (AF) tears. Torsion weakens the intralamellar matrix, compromising disc strength and stiffness, and causing lamellar damage [15]. When combined with bending and compression, these motions can further elevate the risk of disc failure [16]. Recently, we demonstrated that combined extension, lateral bending, and torsion disrupt collagen type II and glycosaminoglycans in the transition zone between the inner AF and nucleus pulposus (NP), cause localized cell death in the inner AF and NP, but maintain the outermost parts viable and structurally intact [2]. However, the study did not include static compression, which is a critical physiological load simulating spinal stress in the standing posture. While static compression of 0.2 MPa was shown to be near‐physiological when combined with torsion, dynamic conditions at 0.6 ± 0.2 MPa amplified the stress on the NP, increasing cell death in the bovine caudal IVD explant model [5]. Finite element modeling has refined 0.1 MPa as a physiologically accurate static load for bovine discs under complex motion [17].

Loading frequency is another critical determinant of disc response in vitro. Higher frequencies increase disc stiffness and reduce phase angle, particularly affecting the poroelastic behavior under flexion/extension and compression [18]. At the cellular level, prolonged exposure to compression and torsion at 1 Hz has been shown to reduce viability [19]. However, low‐frequency complex loading, such as 0.2 Hz [5] or 0.3 Hz [2], has also been shown to be deleterious under complex motion. A potential explanation is that loading was applied asymmetrically in these studies, leading to localized stress concentrations on one side of the disc [4, 20]. Asymmetry has been shown to induce differential molecular responses between the compressed and tensed sides, accelerating degeneration beyond the effects typically associated with symmetric dynamic compression [4]. However, more recent studies challenge this paradigm, demonstrating similar reductions in cell viability and gene expression on both sides of the disc under asymmetrical loading conditions [2, 21].

In this study, we further investigated the interaction between complex spinal motions and specific loading parameters, including magnitude, symmetry, and cycle number, in the progression of disc degeneration. These parameters were examined under combined flexion/extension, torsion, and static compression, a motion that is clinically recognized as a risk factor for disc injury, even at low to moderate frequencies [22].

First, we compared different magnitudes of flexion/extension and torsion under a constant static compression of 0.1 MPa and symmetrical loading at 0.2 Hz frequency. We hypothesized that larger rotational angles of 6° flexion/extension and 4° torsion would induce greater degenerative changes than smaller angles of 3° and 2°, respectively, consistent with our computational model predictions [23] and previous findings at a 0.3 Hz without compression [2]. The second objective was to evaluate the disc response under asymmetrical loading and a tenfold increase in loading cycles at 1 Hz; we hypothesized that this condition would lead to structural failure and distinct effects across the compressed and tensed sides of the disc.

## 2 Materials and Methods

### 2.1 Intervertebral Disc Model

A bovine whole‐organ IVD model (disc diameter: 22.2 ± 1.4 mm) retaining cartilaginous endplates and 7 mm of adjacent bone was prepared from seven freshly slaughtered bovine tails (age: 9 ± 1.3 months; sex: 2 female, 1 male and 4 unknown). Specimen preparation followed a previously described procedure [24], which included the creation of a cross‐shaped notch and a central hole in the bone, subsequent cleaning with a jet lavage system (Pulsavac, Zimmer Biomet, USA) and decontamination in Pen‐Strep solution.

After preparation, samples underwent an initial free swelling phase and were cultured in 30 mL of standard ex vivo bovine IVD culture medium [24] until the start of loading on the next day.

All loaded specimens were obtained from caudal regions with an approximate disc diameter of 22 mm, with slight variations in the vertebral levels. Each experimental group included four IVD samples. For the low‐frequency loading groups, eight discs were harvested from four tails, with two discs from each tail distributed evenly between the groups. The high‐frequency loading group was sourced from three separate tails: one tail provided two samples, and each of the remaining two tails provided one sample. Non‐loaded control samples used for histological analysis were collected from each tail and maintained under free‐swelling conditions until processing on the second day post‐isolation (day 2 controls). Additional non‐loaded controls for gene expression studies were prepared using three separate tails (age: 10.7 ± 1.9 months; disc diameter: 23.7 ± 1.6 mm; sex: 2 male and 1 female) and processed immediately after isolation (day 0 controls).

### 2.2 Loading Protocol

Disc specimens were assembled under sterile conditions using mechanical interfaces and a specimen holder secured with screws and then placed in a chamber specifically designed for multiaxial loading [24]. During assembly, the specimens were hydrated with culture medium and subsequently submerged in 46–50 mL of medium within the chamber before mounting onto the multiaxial bioreactor.

**FIGURE 1:**
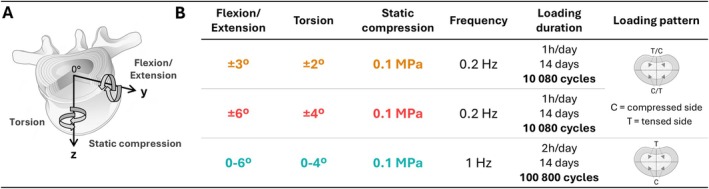
(A) The spine motion simulated in the multiaxial bioreactor, combining flexion/extension, torsion, and static compression. (B) The table shows the three loading groups with parameters applied in this study. The loading pattern for the first two groups involved symmetrical torsion and bending, the latter alternately simulating flexion and torsion (anterior compression and posterior tension) and extension and torsion (anterior tension and posterior compression). The third group was subjected to asymmetrical flexion and torsion to simulate recurring anterior compression and posterior tension.

Specimens were subjected to distinct loading protocols as follows: (group 1) Flexion/Extension ±3°, Torsion ±2°, Static compression 0.1 MPa; (group 2) Flexion/Extension ±6°, Torsion ±4°, Static compression 0.1 MPa; (group 3) Flexion 0°–6°, Torsion 0°–4°, Static compression 0.1 MPa (Figure 1). All groups were loaded for 14 days. Groups 1 and 2 were subjected to 1 h of daily loading at 0.2 Hz, resulting in a total of 10 080 loading cycles. In these groups, torsion and flexion/extension were applied symmetrically around the z‐ and y‐axes, where bending was alternately simulating flexion (anterior compression and posterior tension) and extension (anterior tension and posterior compression) (Figure 1). Group 3 underwent 2 h of daily loading at 1 Hz, accumulating 100 800 cycles. For this group, flexion/extension was applied asymmetrically to target anterior disc regions, simulating recurring anterior compression and posterior tension, while ensuring all loading motions remained in phase.

Discs were continuously maintained in the culture medium at 37°C, either within the bioreactor during loading or in an incubator following loading sessions. The medium was replaced every 2–3 days, and the volume was recorded during each change. Between the 1–2 h loading cycles, a filtered opening on the chamber lid ensured appropriate humidity, CO_2_ level, and gas exchange.

### 2.3 Data and Sample Collection

Disc height changes were monitored daily using a custom‐designed laser‐based measurement system. The height change following loading was calculated as the difference between the heights recorded before and after loading on the same day. The height change after free‐swelling recovery was determined as the difference between the height before loading and the height measured before the previous day's loading session. Relative disc height changes were normalized to the disc height manually measured at the end of the experiment, defined as the distance between the deepest points of the cartilaginous endplates during tissue processing.

The culture medium was collected daily using a syringe after both the loading and swelling phases. A volume of 0.5 mL medium was stored at −20°C for glycosaminoglycan (GAG) and nitric oxide (NO) measurements, while an additional 0.5–1 mL was snap‐frozen and preserved at −70°C for ELISA‐based analyses.

At the 15‐day endpoint, discs were harvested following an overnight recovery period. Each specimen was sagittally cut through the center. For the asymmetrically loaded group, discs were cut to encompass compressed and tensed regions of the disc. One sagittal half was further divided transversely through the disc center and snap‐frozen for histological analysis. The other sagittal half was processed without endplates and bone. The AF of the asymmetrically loaded group was further divided into compressed and tensed regions. Tissue samples ranging from 100 to 200 mg were immediately processed for RNA isolation.

### 2.4 Histological Analysis

Transverse cryosections, 10 μm thick, were prepared and fixed in 100% methanol for general staining or in methanol with 3% hydrogen peroxide (Sigma‐Aldrich, Merck, Germany) for immunohistochemistry. For cell viability analysis, unfixed sections were stained using the lactate dehydrogenase and ethidium homodimer‐1 method [24]. GAGs were visualized using the standard protocol for safranin‐O and fast green staining, and collagen types I and II were stained with previously described protocols for immunohistochemistry [2].

Stained sections were imaged using an Olympus microscope (Tokyo, Japan) and analyzed in ImageJ software. Cell viability and matrix composition were quantified across four distinct disc regions, as described in a prior multiaxial bioreactor study [2]. Briefly, picrosirius red‐stained sections were used to delineate the AF based on its lamellar structure. This delineation was then applied to sections stained for collagen types I and II and GAG to identify the AF and segment it into outer and inner regions. The adjacent tissue beyond the lamellar structure was defined as transitional NP, while the remaining NP tissue was designated as central NP. GAG, collagen type I, and collagen type II were quantified as percentages of positively stained areas in individual disc regions, and the cell viability was expressed as the ratio of viable cells to the total cell count.

Tissue damage was quantified in the outer AF on safranin‐O/fast green‐stained sections, separately in the compressed and tensed regions, by dividing the outer AF into two halves (Figure S1A). In non‐loaded control samples, damage was assessed across the entire outer AF. Images were converted to greyscale, and total tissue damage, defined as voids or discontinuities in the matrix, was highlighted and measured as a percentage of the total tissue area (Figure S1B). Specific damage features, namely transverse intralamellar fissures (i.e., perpendicular to collagen fiber orientation) and interlaminar separations (i.e., delaminations), were manually outlined using the free‐hand selection tool, and their areas were measured and expressed as a percentage of the total damage area.

For both matrix composition and damage quantification, additional data from the eight control specimens used in the previous study [2] were included. Those samples were processed identically to the samples in the present study.

### 2.5 Medium Analysis

GAG release in the culture medium was quantified using the 1,9‐dimethyl‐methylene blue assay. NO release was measured using the Griess Reagent System (Promega, USA; #G2930), and interleukin‐6 (IL‐6) and interleukin‐8 (IL‐8) releases were quantified using bovine‐specific ELISA kits (Kingfisher Biotech, USA; #DIY0670B‐004 and DIY1028B‐004, respectively). Final concentrations were normalized to the medium volume present in the chamber at each time point and to the disc volume. The disc volume was calculated from disc diameter and height, assuming a regular cylindrical shape. Cumulative releases were calculated by summing the daily releases recorded after both the loading and free‐swelling phases, starting from the first media change.

### 2.6 RNA Isolation and Gene Expression Analysis

RNA was isolated according to previously described protocols [2, 25]. Real‐time quantitative polymerase chain reaction (qPCR) was performed with 4 ng/μL of cDNA using a TaqMan Universal Master Mix (Applied Biosystems, USA). Target genes encoding matrix metalloproteinases (MMP1, MMP3, MMP9, MMP13), a disintegrin and metalloproteinase with thrombospondin motifs (ADAMTS4, ADAMTS5), interleukins (IL‐1β, IL‐6), tumor necrosis factor‐alpha (TNF‐α), collagen types I and II (COL1, COL2), aggrecan (ACAN), and cartilage oligomeric matrix protein (COMP) were analyzed with custom‐designed primers (Table S1). Ribosomal protein lateral stalk subunit P0 (RPLP0), MMP19, IL‐8, and elastin (ELN) were analyzed using gene expression assays (Life Technologies, USA). Relative gene expression was determined using the comparative Ct method (ΔΔCt), with RPLP0 as the endogenous control and the average gene expression value of day 0 non‐loaded control samples as the reference.

### 2.7 Statistical Analysis

Statistical analysis was performed using GraphPad Prism 9 software (GraphPad, USA). Data normality was evaluated using the Shapiro–Wilk test. For comparison between two groups, an unpaired parametric *t*‐test with Welch's correction was applied to data with normal distribution and comparable standard deviations, while the unpaired non‐parametric Kolmogorov–Smirnov test was used for non‐normally distributed data. Three or more loading groups were assessed using one‐way ANOVA with Tukey's multiple comparisons test or non‐parametric Kruskal‐Wallis test with Dunn's multiple comparisons test. To evaluate the combined effects of loading conditions and time, a repeated measures two‐way ANOVA with the Geisser–Greenhouse correction and Tukey's multiple comparisons test was conducted. Statistical significance was defined as a *p*‐value less than 0.05. Technical replicates were averaged, and biological replicates were shown either as individual points or as the mean and standard deviation of four samples.

## 3 Results

### 3.1 Disc Height Changes

**FIGURE 2:**
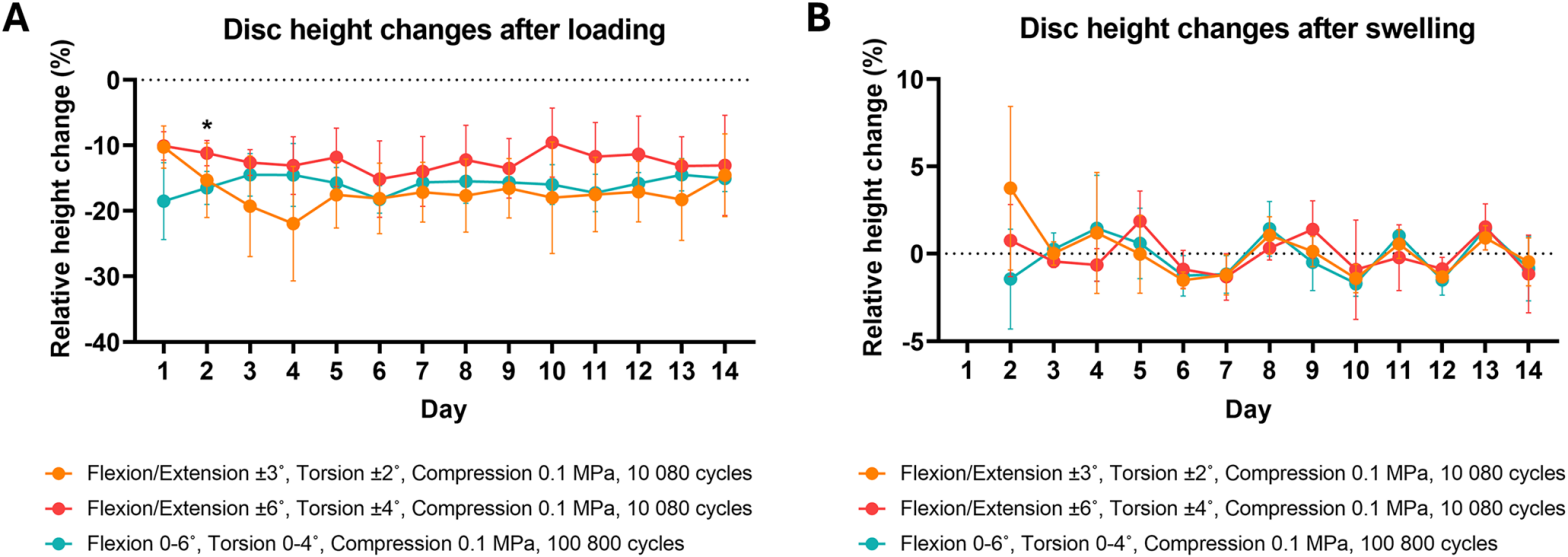
Disc height changes were measured daily over 14 days, following loading and free‐swelling recovery. (A) Changes in height following loading were calculated relative to the pre‐loading height recorded on the same day. (B) Measurements of height after swelling were first recorded on day 2, after the recovery from initial loading on day 1. Changes in height following swelling were calculated relative to the pre‐loading height recorded the previous day. Data show mean values from 4 samples and standard deviation. A repeated‐measure two‐way ANOVA test was performed to analyze the effects of loading parameters and time on height changes, where *p* < 0.05 (*) was statistically significant between the red and green groups.

The average disc height loss after loading and subsequent recovery during swelling was consistent across groups (Figure 2). On day 2, height loss was more pronounced in discs subjected to asymmetrical loading and greater loading cycles compared to the group subjected to lower loading cycles and greater angles. On average, the loss was −17.1% ± 5.8% for the low‐angle and low‐cycle group, −12.3% ± 4.5% for the high‐angle and low‐cycle group, and −16% ± 2.9% for the asymmetrically loaded, high‐cycle group.

### 3.2 Cell Viability

**FIGURE 3:**
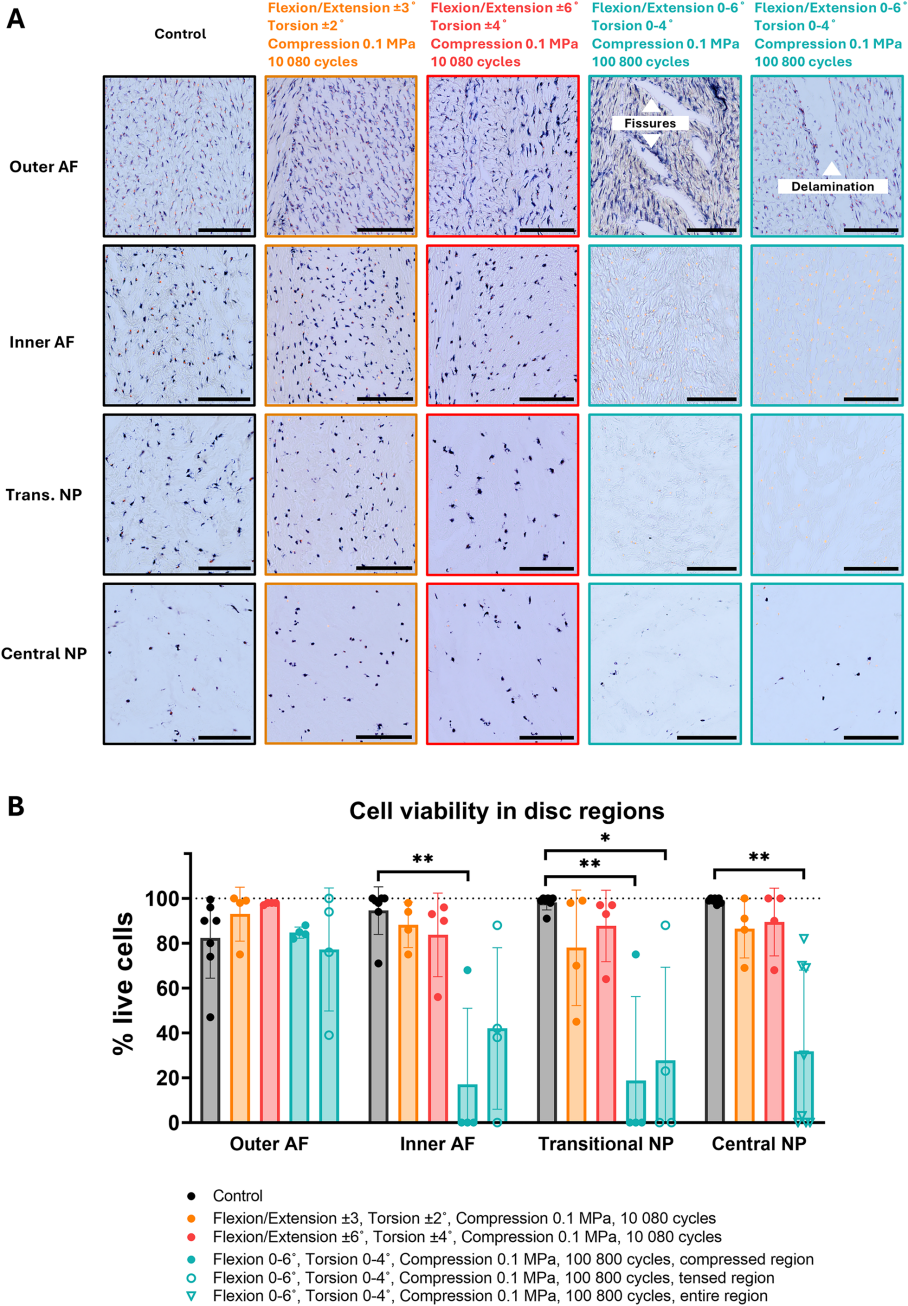
(A) Representative sections from specimens subjected to different loading conditions and a non‐loaded control, stained with lactate dehydrogenase and ethidium homodimer to assess cell viability. Live cells appear as blue or blue/orange, and dead cells are orange. Images show the viability in outer and inner regions of annulus fibrosus (AF) and transitional and central nucleus pulposus (NP). The group loaded asymmetrically and with higher cycle number (green) is represented with two specimens to highlight high viability around fissures and delaminations in the tensed and compressed regions, respectively. Scale bar: 200 μm. (B) Quantitative analysis in designated disc regions. In the asymmetrically loaded group (green), the AF and transitional NP were analyzed separately on the compressed and tensed sides, and NP was assessed across the whole region. Data represent individual data points and mean ± standard deviation for control (*n* = 7) and loaded (*n* = 4) specimens. Statistical comparisons within each region across groups were conducted using one‐way ANOVA or the Kruskal‐Wallis test, where *p* < 0.01 (**) and *p* < 0.001 (***) were statistically significant.

Cell viability remained high across all disc regions in groups subjected to a lower number of loading cycles (Figure 3A,B). Discs exposed to asymmetrical loading and higher cycle numbers retained high cell viability in the outermost AF, including areas adjacent to fissures and delaminations (Figure 3A). In contrast, significant decreases in viability were observed in the inner AF, transitional NP, and central NP compared to donor‐matched controls: −73.8% ± 40.9%, −78% ± 35.8%, −72.8% ± 27%, respectively, on the compressed side, and −48.8% ± 41.7%, −69% ± 39.2% and −60.3% ± 37.9% on the tensed side. The decline was more pronounced on the compressed side than the tensed side of the inner AF. Although the group loaded asymmetrically and with higher cycles showed a trend toward reduced viability in the inner AF and transitional NP relative to the groups loaded symmetrically and with lower cycles, these differences did not reach statistical significance due to an outlier specimen with a high cell viability in the asymmetric group.

### 3.3 Structural Alterations in the Outer AF

**FIGURE 4:**
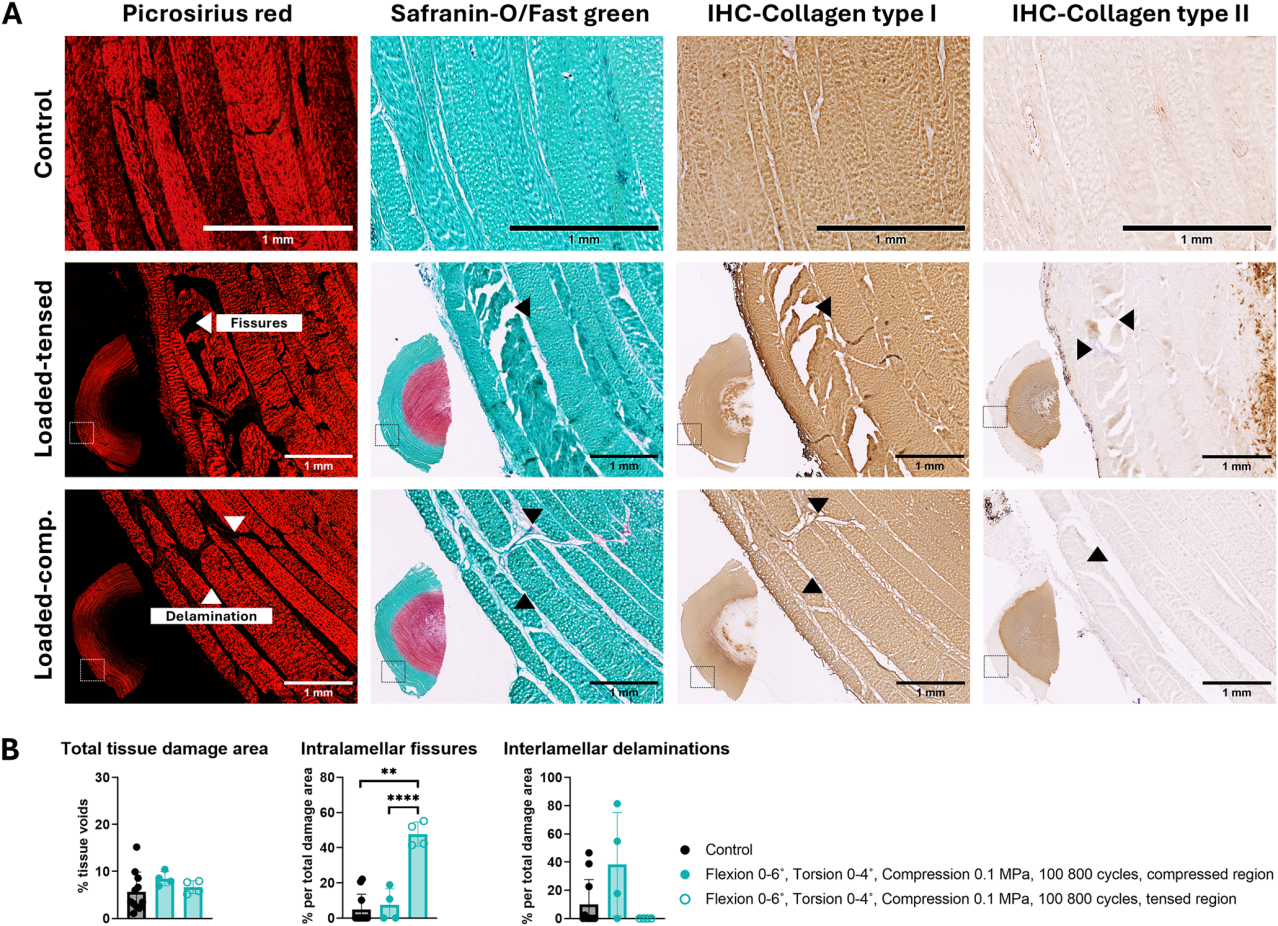
(A) Structural changes in the outer annulus fibrosus (AF) were observed in the group subjected to asymmetrical loading and higher loading cycles, shown in comparison to a non‐loaded control. Consecutive sections were analyzed using multiple staining techniques: Picrosirius red to visualize lamellar structure, safranin‐O/fast green staining to differentiate glycosaminoglycan (GAG) content (red) and collagen fibers (green), and immunohistochemistry (IHC) to detect positive brown signals of collagen types I and II. Two representative disc specimens from the loaded group are shown, including macroscopic views and high‐magnification images highlighting fissures in the tensed region and delaminations in the compressed region. Leftward‐pointing arrows indicate intralamellar fissures oriented perpendicular to the collagen fiber orientation; the rightward‐pointing arrow indicates cell accumulation near fissures; the upward‐pointing arrow denotes interlaminar separations between parallel‐aligned annular fibers; and the downward‐pointing arrow highlights a fissure associated with fibrous and GAG deposition. (B) Quantitative analysis of tissue damage was performed on safranin‐o/fast green‐stained sections, measuring total damage area and specific contributions from fissures and delaminations in control discs (*n* = 11) and in compressed and tensed regions of loaded specimens (*n* = 4). Data represent individual data points and mean ± standard deviation. Statistical analysis between groups was performed using one‐way ANOVA or the Kruskal‐Wallis test, where *p* < 0.01 (**) and *p* < 0.0001 (****) were statistically significant.

In contrast to non‐loaded controls and groups subjected to symmetrical loading at lower loading cycles, which maintained normal disc structure, asymmetrically loaded discs exposed to higher loading cycles exhibited distinct macroscopical alterations in the outermost regions of the AF. These changes included intralamellar fissures oriented perpendicular to the collagen fiber direction and/or interlaminar separations (i.e., delaminations) (Figure 4A, leftward and upward arrows, respectively). To eliminate the possibility of cutting artifacts caused by sectioning, the findings were macroscopically validated across four consecutive tissue sections using multiple histological stains and further quantitatively assessed on safranin O/fast green‐stained sections.

Quantitative analysis confirmed the presence of tissue damage in both non‐loaded control and loaded samples; however, specific structural alterations were statistically more prevalent in the loaded samples than in controls (Figure 4B). Intralamellar fissures occurred more significantly on the tensed side of the disc, while delaminations were predominantly found on the compressed side, although with inter‐sample variability. Delaminations were absent in the tensed regions. Fissures were typically confined to a single lamella, while delaminations spanned two to four lamellae (Figure 4A).

All loaded specimens exhibited at least one of these structural changes. When both fissures and delamination co‐occurred, they were typically located on opposite sides of the disc along the same circumferential plane, with no clear evidence of a causal relationship between them. In some samples, cell accumulation and fibrous matrix or GAG deposition were observed within fissures (Figure 4A, rightward and downward arrows, respectively).

Additional extracellular matrix analysis revealed no significant differences in collagen types I and II or GAG content between loaded and control groups across disc regions (Figure S2).

### 3.4 Molecule Release Into the Medium

**FIGURE 5:**
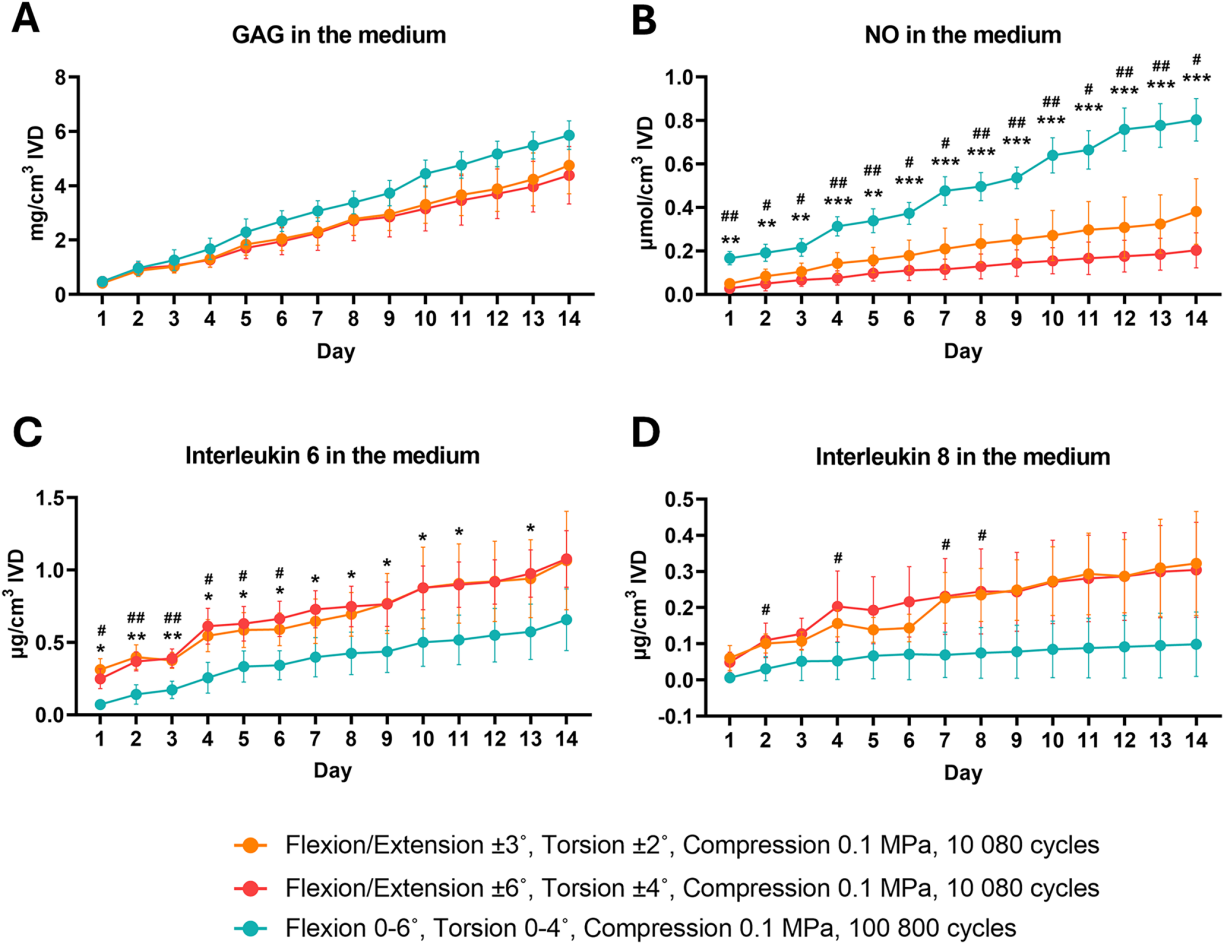
Cumulative release of glycosaminoglycans (GAGs; A), nitric oxide (NO; B), interleukin‐6 (IL‐6; C) and interleukin‐8 (IL‐8; D) in the medium following the combined daily loading and free‐swelling periods across 14 days. Data are presented as mean values from 4 samples and standard deviation. A repeated measure two‐way ANOVA was performed to analyze the effects of loading parameters and time, where *p* < 0.05 (*/#), *p* < 0.01 (**/##) and *p* < 0.001 (***/###) were statistically significant. The hash symbol (#) indicates a statistical difference between the orange and green groups, while the asterisk (*) symbol indicates a statistical difference between the red and green groups.

The daily release of GAG into the medium was consistent across all loaded groups, with a trend toward higher release observed in the group subjected to asymmetrical loading and a higher number of loading cycles (Figure 5A). This group also released significantly more NO into the medium compared to the groups exposed to symmetrical loading and fewer loading cycles on all loading days (Figure 5B). In contrast, the group subjected to asymmetric loading and a higher number of loading cycles exhibited lower levels of IL‐6 and IL‐8 release than the groups loaded symmetrically and with fewer loading cycles (Figure 5C,D). These differences were significant for IL‐6 during the first six days in both groups loaded symmetrically at fewer cycles and subsequently for the group exposed to higher rotational angles and fewer loading cycles. For IL‐8, significant differences were observed on loading days 2, 4, 7 and 8 between the symmetrically loaded group subjected to lower rotational angles and cycles and the asymmetrically loaded group subjected to higher rotational angles and cycles.

### 3.5 Gene Expression Analysis

**FIGURE 6:**
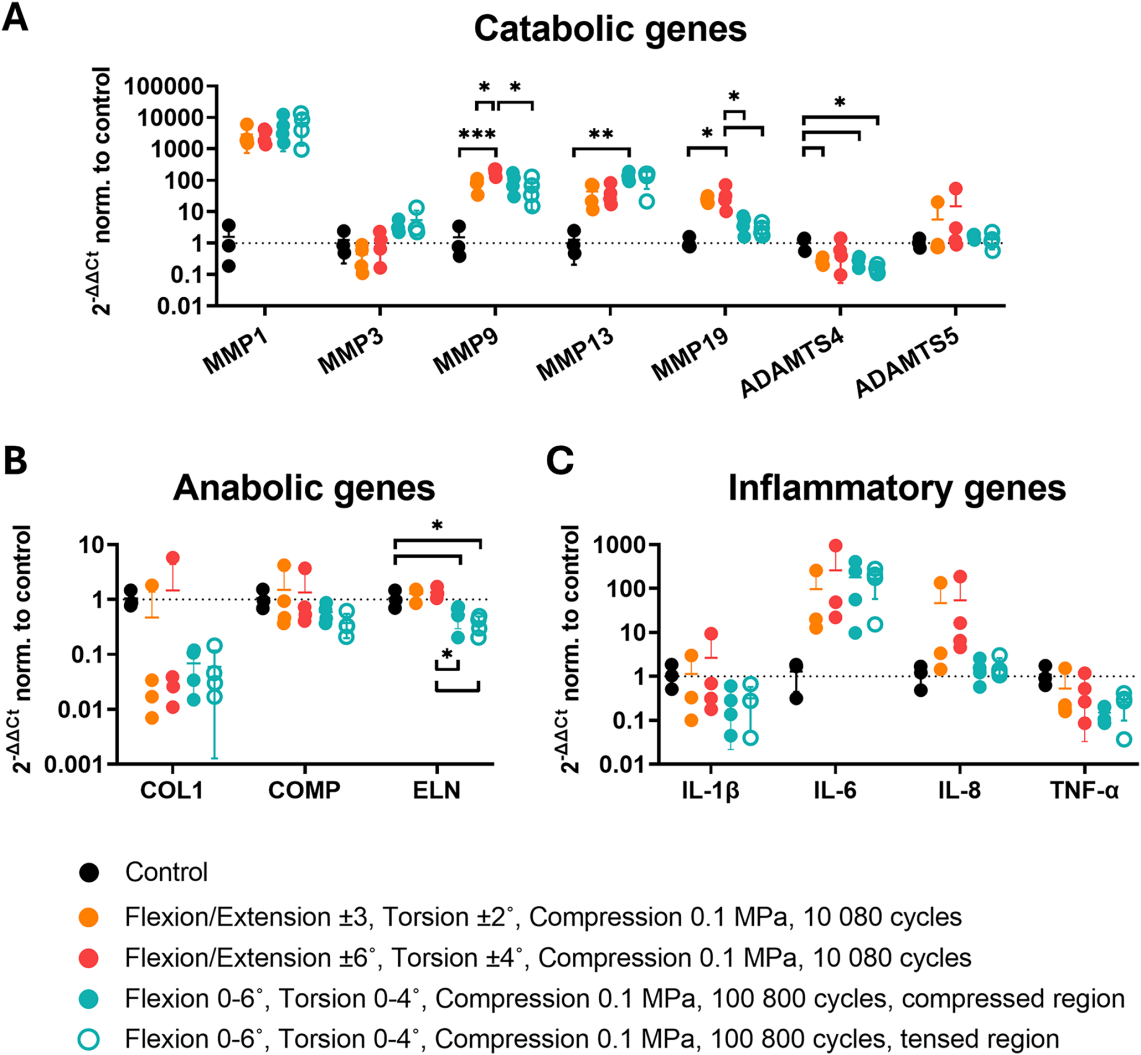
Expression levels of catabolic (A), anabolic (B), and inflammatory (C) genes in the outer annulus fibrosus (AF). In the group exposed to asymmetrical loading and higher loading cycles, gene expression was assessed separately in the compressed and tensed regions of the AF. Gene expression was quantified using the comparative Ct method (ΔΔCt), normalized to an endogenous control gene and day 0 control samples. Data points represent individual samples, presented as the mean ± standard deviation. Statistical comparisons between groups were conducted using one‐way ANOVA or the Kruskal‐Wallis test, where *p* < 0.05 (*), *p* < 0.01 (**), and *p* < 0.001 (***) were statistically significant.

Gene expression analysis was performed in the outer AF on catabolic, anabolic, and inflammatory genes characteristic of a disc degenerative profile. For catabolic genes, the symmetrical low‐angle/low‐frequency, symmetrical high‐angle/low‐frequency, and asymmetrical high‐frequency groups exhibited either a trend toward upregulation or a significant upregulation of MMP1 (2877‐, 2799‐, and 6191‐fold relative to controls, respectively), MMP9 (79‐, 190‐, and 79‐fold), MMP13 (44‐, 41‐, and 126‐fold), and MMP19 (24‐, 34‐, and 4‐fold) (Figure 6A). MMP3 showed this trend only in the asymmetric and high‐loading cycle group. ADAMTS4 was downregulated across all groups, with greater variability in the group loaded symmetrically at higher angles and low cycle numbers. ADAMTS5 expression was comparable to controls.

The expression of the anabolic genes was variable among groups. COL1 showed a downregulation trend, particularly in the high‐cycle group, while the symmetrically loaded low‐cycle groups showed a greater variability, considering outliers. COMP and ELN expressions were similar to controls in symmetrical low‐cycle groups but either showed a downregulation trend or were significantly downregulated in the asymmetrical high‐cycle group, respectively (Figure 6B).

The inflammatory gene IL‐1β displayed expression levels similar to controls in symmetrical low‐cycle groups but showed a trend toward downregulation in asymmetrical high‐cycle groups (Figure 6C). IL‐6 was generally upregulated across all groups (96‐, 261‐, 175‐fold change to controls), while IL‐8 was upregulated in groups subjected to lower cycle numbers. TNF‐α expression showed a downregulation trend in the group exposed to high cycles of asymmetrical loading.

Across all genes, changes in gene expression were similar between the compressed and tensed AF regions within the asymmetrically loaded group. Gene expression analysis of the NP was performed only for the symmetrical low‐cycle groups, as these conditions maintained high cell viability and yielded sufficient RNA for analysis. In these groups, MMP1 expression was significantly upregulated, whereas COL1, COL2, ACAN, and COMP were downregulated (Figure S3).

## 4 Discussion

This study has been built on our previous research using next‐generation bioreactors and further explored how complex and varied loading conditions contribute to detrimental disc changes. We hypothesized that (1) under symmetric loading, high rotational angles would induce more detrimental effects than low rotational angles and (2) asymmetric loading and increased loading cycles would induce regional damage. When applied symmetrically at low cycle numbers, the combination of flexion/extension, torsion, and static compression did not induce significant degenerative changes beyond alterations in gene expression, regardless of rotational angles, which contrasted with our first hypothesis. However, our second hypothesis was confirmed in that a ten‐fold increase in cycle number, applied asymmetrically, resulted in lamellar damage in the outer AF. While the specific structural damage was attributed to either the compressed or tensed disc side, changes in cell viability were similar across disc sides. Notably, viability was markedly reduced in deeper disc regions, whereas the outer AF cells adjacent to annular damage remained highly viable.

Our results are consistent with previous reports indicating that combined flexion, torsion, and compression increase intradiscal pressure, forcing the annular lamellae to bear greater stress and resulting in tears and delaminations [12]. Intralamellar damage was previously observed in discs subjected solely to torsion [15], while radial delaminations within the AF were reported under combined torsion and flexion/extension [26]. In this study, structural alterations are likely driven by the combination of rotational motions with compression, which is known to generate peak shear stresses that compromise both intralamellar and interlamellar integrity, ultimately leading to mechanical failure [11, 16, 27, 28, 29].

Despite structural damage to the AF, height measurements indicated that the NP maintained its compressive and recovery function, suggesting a dissociation between outer structural failure and inner mechanical performance. The similar and reversible height losses across all groups and time points further indicate that these reductions are primarily due to applied compression, which plays a critical role in disc height changes [30, 31], rather than structural damage. However, the average 12%–17% height loss that occurred both with and without structural change exceeds the physiological 10% change [31], indicating a degenerative trajectory that may eventually compromise function [30, 31]. These findings require validation under further increased loading cycles to capture mechanically relevant degeneration, such as herniation.

Previous studies have implicated mechanical injuries as key initiators of disc degeneration [32, 33]. Although this study suggests a role of structural alterations and cellular response, it could not establish a causal link between them, as these effects did not occur uniformly. Notably, high cell viability observed near fissures and delaminations suggests that mechanical strains are region‐specific and depend on local structure and composition. Shear stresses may therefore have contributed independently or concurrently to structural failure in the outer AF and cell death in the inner AF and NP regions [34], the latter being particularly sensitive to mechanical strain [35]. Indeed, complex loading has been associated with pronounced cellular death in both AF and NP compared to simplified uniaxial loading [3, 36]. However, it remains unclear whether one type of failure predisposes the tissue to experience other degenerative changes. Dynamic tracking of these degeneration characteristics over shorter culture periods would help clarify the cause‐and‐effect relationship between structural integrity and cellular health [32, 33].

In this study, we demonstrate the contribution of structural changes while excluding the role of extracellular matrix disintegration. This contrasts with complex motion involving only rotational loading, which has been shown to cause GAG and collagen type II loss [2]. Interestingly, such matrix degradation was not observed in this study despite a greater number of loading cycles and upregulation of catabolic gene expression. These different outcomes between studies highlight the distinct effects of specific loading conditions, such as motions or loading parameters, on disc structure and composition, possibly influenced by the presence or absence of compressive forces. Indeed, finite element analysis confirmed that, without compression, disc failure in the AF is unlikely to occur from repetitive bending alone [27]. Interestingly, compression appears to play a lesser role in mediating cellular response, as patterns of cell death in the inner AF and NP were comparable in studies with and without compression. Together, these findings suggest that modulating compressive forces rather than torque alone could be a strategic approach to generate region‐specific degenerative profiles that encompass both structural and molecular alterations.

Identifying optimal loading durations and frequencies for complex motions is critical for understanding the mechanisms driving degeneration. The structural changes observed in the present study occurred after approximately three times more loading cycles than the compositional changes in the rotational loading study. Previous research also associated higher loading rates with increased disc failure [37, 38] and greater cell death in NP [19]. This study confirmed that, over the same experiment duration, fewer loading cycles with shorter and less frequent loading (1 h/day, 0.2 Hz; 10 080 cycles) under symmetrical flexion/extension maintained better cell viability, reduced GAG and NO release, and preserved disc structural integrity compared to longer, more frequent loading (2 h/day, 1 Hz; 100 800 cycles) under asymmetrical conditions. Of note, these observations do not allow any conclusions regarding the single influence of loading frequency, duration, and symmetry. Interestingly, IL‐6 and IL‐8 releases were more pronounced in groups subjected to fewer cycles and symmetrical loading, suggesting an acute inflammatory response enabled by high cell viability. In contrast, discs with altered structure and reduced viability had a lower cytokine response, suggesting a chronic degenerative state in which the typical acute inflammatory cytokine response was no longer possible.

Despite the difference in symmetry and cycle number, groups subjected to symmetric and lower loading cycles showed similar upregulation of catabolic genes compared to the asymmetrically loaded high‐cycle group, consistent with findings from studies involving combined torsion and compression [19]. However, differential expression of specific markers, such as MMP19 and ELN, points to potential region‐specific or load‐specific responses. As this study and our previous work represent the first gene expression datasets in response to multiaxial loading, further research is needed to elucidate the early molecular responses to prolonged complex loading in ex vivo models and to determine whether these responses contribute to disc degeneration.

Contrasting our first hypothesis, increasing motion magnitude did not induce a more significant disc response. This is likely because the two groups compared at different magnitudes were subjected to low cycle numbers and uniform loading. These results also suggest that moderate increases of 3° in flexion/extension and 2° in torsion may be insufficient to induce measurable changes when applied symmetrically at low cycle numbers. Instead, such protocols may help maintain a near‐physiological state and serve as suitable controls in future multiaxial bioreactor studies.

In line with our second hypothesis, the more severe outcomes observed may be attributed to the high cycle number under asymmetric loading. Due to the experimental design, we are unable to distinguish between the effects of loading frequency, duration, and symmetry, and further studies are required to dissect the contributions of these individual parameters. Asymmetric loading has been shown to adversely affect cell viability and gene expression [2, 4, 21] and predispose to failure and tearing, particularly on the opposing side of a flexed disc [39]. Consistent with this, intralamellar fissures in the asymmetric, high‐cycle group were more frequent on the tensed side, whereas delaminations were more prevalent on the compressed side, although the distribution of the latter varied across samples. Despite this localized structural disruption, both sides of the disc showed comparable reductions in viability and gene expression, supporting the concept of a whole‐organ mechanical response to complex loading, as also observed in our previous rotational loading study [2]. Integrating these findings with our previous research suggests that asymmetric flexion/extension and torsion combined with static compression exposes the disc to structural failure through fissures and delaminations.

One limitation of this study concerns the sample size. The analysis of alterations was limited by the number of available bioreactors, which constrained the sample size for simultaneous mechanical loading. Furthermore, whole organ evaluation beyond histology could provide a more comprehensive understanding of the spatial distribution of structural changes across the entire disc. Spatial transcriptomics would further clarify molecular changes on the regional level. Moreover, several observed effects were restricted to a single group subjected to high cycles of asymmetric loading, limiting the ability to independently evaluate the contributions of loading magnitude, symmetry, frequency, and duration. While high cycles of asymmetric loading conditions increased the risk of strain‐induced cellular failure, it remains unclear whether such failure precedes or follows structural damage. Considering the preserved cell viability in the outer AF, long‐term post‐loading culture could determine whether structural damage is reversible through repair processes. Using a less heterogeneous group of donors would help clarify the influence of age and sex on biological variability and whether the disc diameter affects the likelihood of structural alterations. More accurate cross‐sample comparisons would also require normalization of mechanical inputs, specifically by applying consistent torque rather than rotational angles. Since the current bioreactor setup only allows adjustment of rotation angles, future studies should either calibrate angles to standardize torque or incorporate disc size into computational models to guide angle selection.

Our observation that structural damage and cell death do not co‐localize suggests that, in early disc degeneration, and particularly in the absence of substantial structural disruption, mechanical strain may primarily act through perturbation of molecular pathways. These pathways likely involve altered mechanosignalling, changes in gene expression and protein release, and ultimately apoptosis or senescence. These findings highlight the importance of strategies to reduce mechanical stress, enhance cellular resilience, and preserve matrix integrity to slow degenerative progression and maintain disc function. They also support the development of personalized therapeutic approaches tailored to region‐specific disc vulnerability, including mechanical load modulation, targeted cellular and molecular therapies, and localized drug delivery.

## Author Contributions

**Amra Šećerović:** conceptualization, methodology, validation, formal analysis, investigation, visualization, writing‐original draft, writing‐revision. **Aapo Ristaniemi:** conceptualization, methodology, writing‐revision. **Francesco Crivelli:** conceptualization, methodology, software. **Sarah Heub:** conceptualization, resources, funding acquisition. **Mauro Alini:** conceptualization, resources, funding acquisition. **Gilles Weder:** conceptualization, resources, funding acquisition. **Diane Ledroit:** conceptualization, methodology, validation, project administration. **Stephen J. Ferguson:** conceptualization, methodology, funding acquisition, writing‐revision. **Sibylle Grad:** conceptualization, methodology, resources, funding acquisition, project administration, writing‐revision.

## Funding

This work was supported by the Swiss National Science Foundation under grant number 189915, the AO Foundation, and AO Spine.

## Conflicts of Interest

Sibylle Grad and Mauro Alini are Editorial Board members of JOR Spine; Sibylle Grad is the corresponding author and Mauro Alini is co‐author of this article. To minimize bias, they were excluded from all editorial decision‐making related to the acceptance of this article for publication.

## Supporting information


**Figure S1:** Quantification method for structural changes in the intervertebral disc.

**Figure S2:** Quantitative analysis of collagen types I and II, and glycosaminoglycans (GAGs) in the outer and inner regions of the annulus fibrosus (oAF and iAF, respectively), and the transitional and central regions of the nucleus pulposus (tNP and cNP, respectively).

**Figure S3:** Expression levels of catabolic, anabolic, and inflammatory genes in the nucleus pulposus in low‐angle/low‐frequency and high‐angle/low‐frequency groups.

**Table S1:** Custom‐designed bovine primer sequences (forward [F], reverse [R], probe [P]) with 5′ FAM and 3′ TAMRA modification, and bovine gene expression assays of commercially available primers.

## Data Availability

The data that support the findings of this study are available from the corresponding author upon reasonable request.
