# Supplementary material for: Combined Flexion, Torsion and Compression Drive Distinct Intervertebral Disc Failure Mechanisms Under Asymmetric, High‐Cycle Loading
Source: JOR Spine. 2026 Feb 11;9(1):e70163. doi: 10.1002/jsp2.70163 (PMC12892121; doi:10.1002/jsp2.70163)
Supplement: Supplementary file 1 — Figure S1: Quantification method for structural changes in the intervertebral disc. [file JSP2-9-e70163-s002.docx]

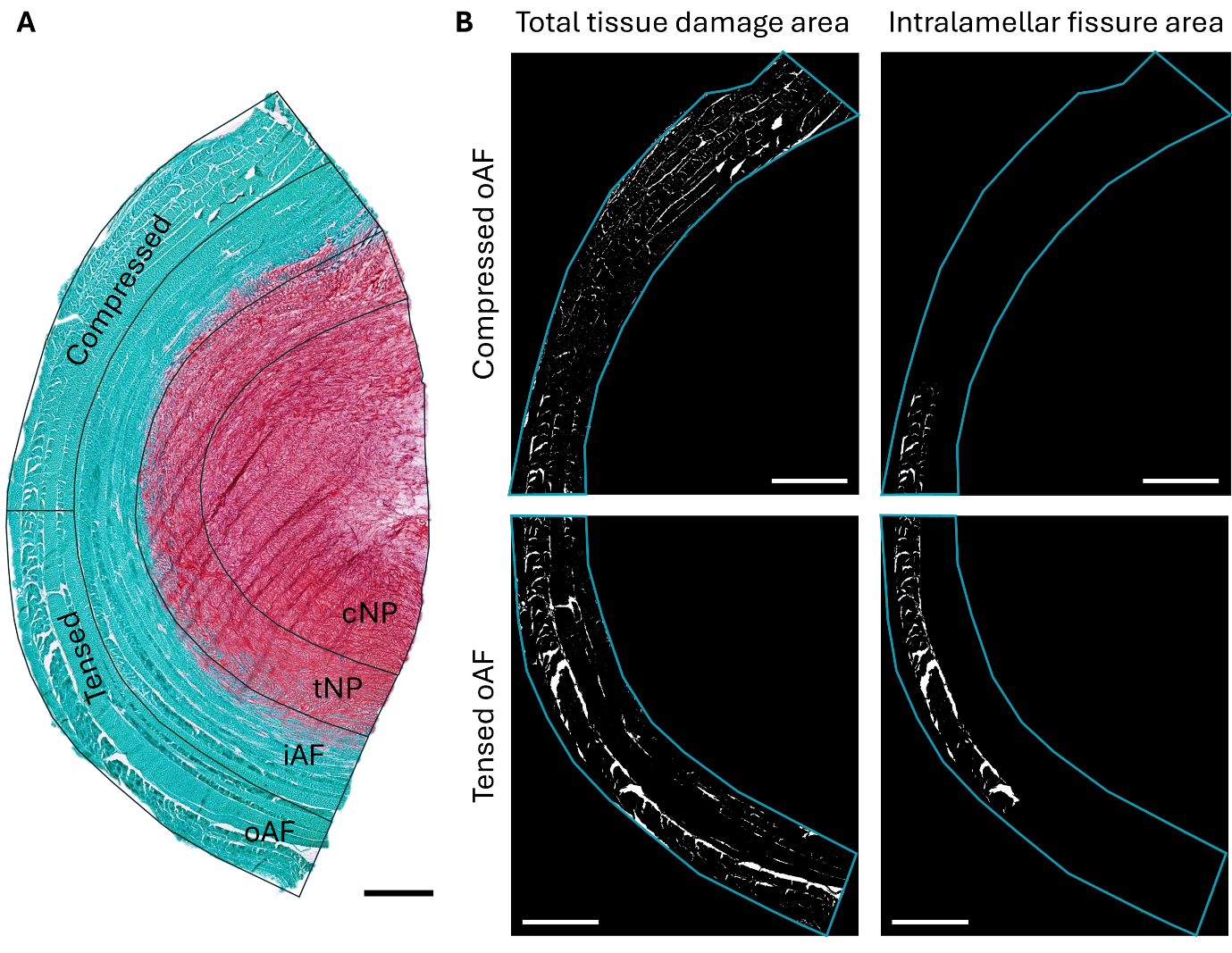


***Supp. Fig. 1.*** *Quantification method for structural changes in the intervertebral disc. (A) Representative disc section showing delineated regions of the outer annulus fibrosus (oAF), inner annulus fibrosus (iAF), transitional nucleus pulposus (tNP), and central nucleus pulposus (cNP). Outer AF is divided to highlight compressed and tensed areas. The section is stained with a combination of safranin-O and fast green to visualize glycosaminoglycan and fibrous collagen content, respectively. (B) Isolated regions of compressed and tensed outer AF are highlighted in blue, with areas of complete tissue damage and intralamellar fissures marked in white, and positively stained matrix indicated in black. Tissue damage was measured as a percentage of the total tissue area using ImageJ, while the intralamellar fissure area was expressed as a percentage of the total damaged area. Scale bar = 2 mm.*
