# Supplementary material for: Combined Flexion, Torsion and Compression Drive Distinct Intervertebral Disc Failure Mechanisms Under Asymmetric, High‐Cycle Loading
Source: JOR Spine. 2026 Feb 11;9(1):e70163. doi: 10.1002/jsp2.70163 (PMC12892121; doi:10.1002/jsp2.70163)
Supplement: Supplementary file 2 — Figure S2: Quantitative analysis of collagen types I and II, and glycosaminoglycans (GAGs) in the outer and inner regions of the annulus fibrosus (oAF and iAF, respectively), and the transitional and central regions of the nucleus pulposus (tNP and cNP, respectively). [file JSP2-9-e70163-s003.docx]

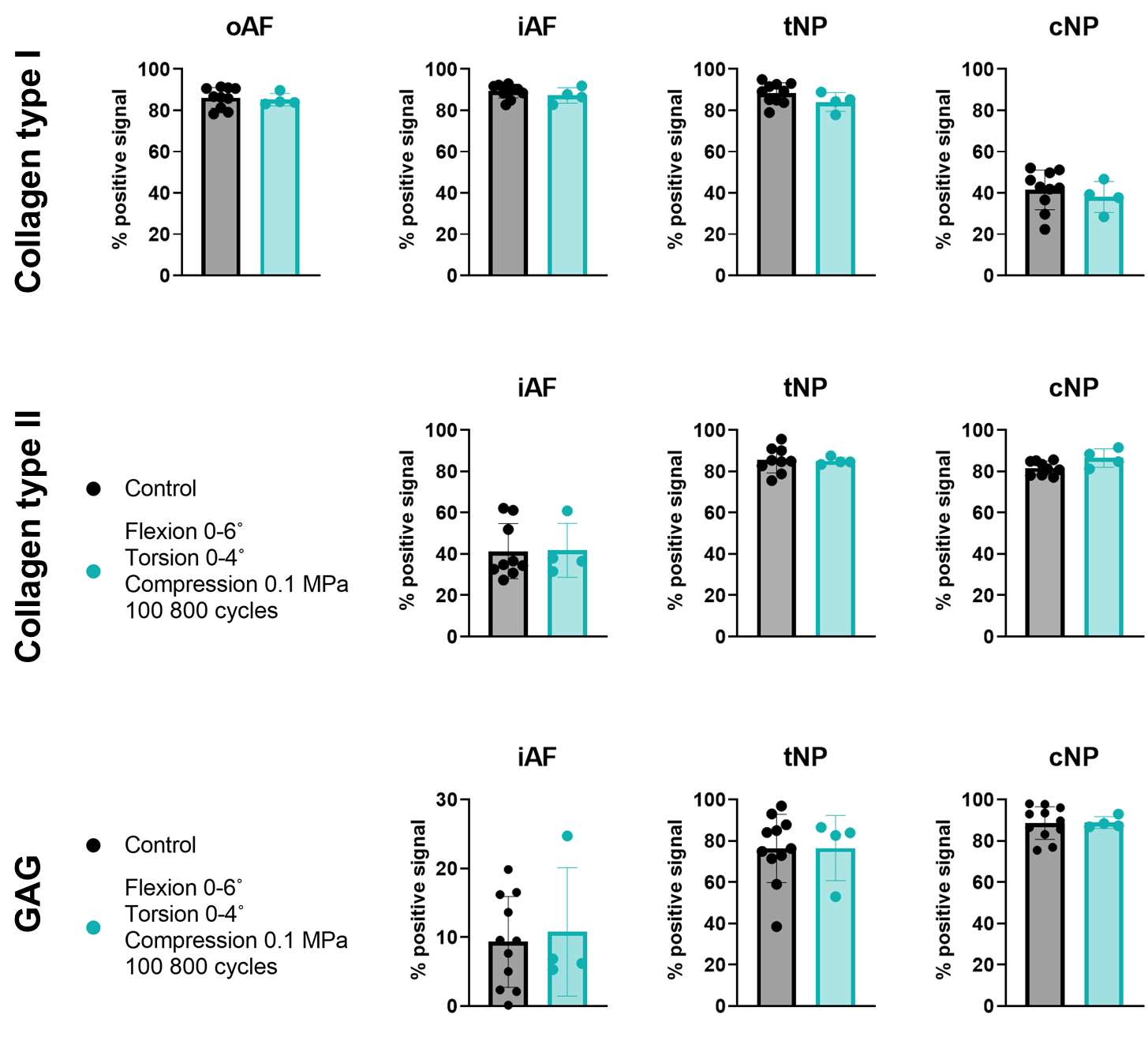


***Supp. Fig. 2.*** *Quantitative analysis of collagen types I and II, and glycosaminoglycans (GAGs) in the outer and inner regions of the annulus fibrosus (oAF and iAF, respectively), and the transitional and central regions of the nucleus pulposus (tNP and cNP, respectively). Tissue sections from loaded (n = 4) and control (n = 11) groups were analysed. Statistical comparisons between groups were performed using the parametric t-test with Welch’s correction and the non-parametric t-test.*
