# Supplementary material for: Combined Flexion, Torsion and Compression Drive Distinct Intervertebral Disc Failure Mechanisms Under Asymmetric, High‐Cycle Loading
Source: JOR Spine. 2026 Feb 11;9(1):e70163. doi: 10.1002/jsp2.70163 (PMC12892121; doi:10.1002/jsp2.70163)
Supplement: Supplementary file 3 — Figure S3: Expression levels of catabolic, anabolic, and inflammatory genes in the nucleus pulposus in low‐angle/low‐frequency and high‐angle/low‐frequency groups. [file JSP2-9-e70163-s004.docx]

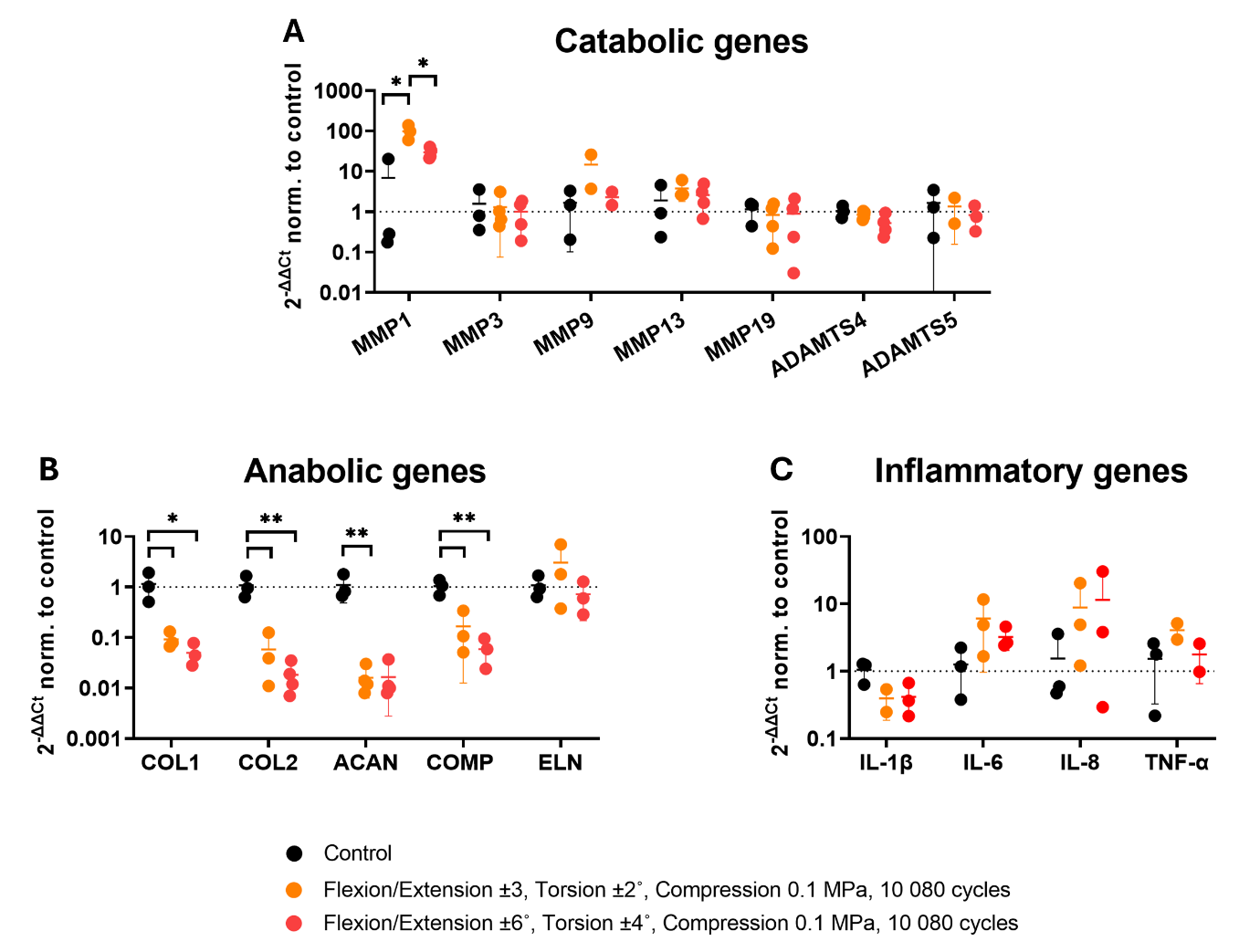


***Supp. Fig. 3.*** *Expression levels of catabolic (A), anabolic (B), and inflammatory (C) genes in the nucleus pulposus in low-angle/low-frequency and high-angle/low-frequency groups. Gene expression was quantified using the comparative Ct method (ΔΔCt), normalized to an endogenous control gene and day 0 control samples. Data points represent individual samples, presented as the mean ± standard deviation. Statistical comparisons between groups were conducted using one-way ANOVA or the Kruskal-Wallis test, where p< 0.05 (*) and p< 0.01 (**) were statistically significant.*
