# Supplementary material for: Combined Flexion, Torsion and Compression Drive Distinct Intervertebral Disc Failure Mechanisms Under Asymmetric, High‐Cycle Loading
Source: JOR Spine. 2026 Feb 11;9(1):e70163. doi: 10.1002/jsp2.70163 (PMC12892121; doi:10.1002/jsp2.70163)
Supplement: Supplementary file 4 — Table S1: Custom‐designed bovine primer sequences (forward [F], reverse [R], probe [P]) with 5′ FAM and 3′ TAMRA modification, and bovine gene expression assays of commercially available primers. [file JSP2-9-e70163-s001.docx]

***Supp. Table 1.*** *Custom-designed bovine primer sequences (forward [F], reverse [R], probe [P]) with 5' FAM and 3' TAMRA modification, and bovine gene expression assays of commercially available primers.*

| **Genes** | **Primer sequences** |
| --- | --- |
| ACAN | (F) 5'-CCA ACG AAA CCT ATG ACG TGT ACT-3'  (R) 5'-GCA CTC GTT GGC TGC CTC-3'  (P) 5'-ATG TTG CAT AGA AGA CCT CGC CCT CCA T-3' |
| ADAMTS4 | (F) 5'-CCC CAT GTG CAA CGT CAA G-3'  (R) 5'-AGT CTC CAC AAA TCT GCT CAG TGA-3'  (P) 5'-AGC CCC CGA AGG GCT AAG CGC-3' |
| ADAMTS5 | (F) 5'-GAT GGT CAC GGT AAC TGT TTG CT-3'  (R) 5'-GCC GGG ACA CAC CGA GTA C-3'  (P) 5'-AGG CCA GAC CTA CGA TGC CAG CC-3' |
| COL1 A2 | (F) 5'-TGC AGT AAC TTC GTG CCT AGC A-3'  (R) 5'-CGC GTG GTC CTC TAT CTC CA-3'  (P) 5'-CAT GCC AAT CCT TAC AAG AGG CAA CTG C-3' |
| COL2 A1 | (F) 5'-AAG AAA CAC ATC TGG TTT GGA GAA A-3'  (R) 5'-TGG GAG CCA GGT TGT CAT C-3'  (P) 5'-CAA CGG TGG CTT CCA CTT CAG CTA TGG-3' |
| COMP | (F) 5'-CCA GAA GAA CGA CGA CCA GAA-3'  (R) 5'-TCT GAT CTG AGT TGG GCA CCT T-3'  (P) 5'-ACG GCG ACC GGA TCC GCA A-3' |
| IL-1β | (F) 5'-TTA CTA CAG TGA CGA GAA TGA GCT GTT-3'  (R) 5'-GGT CCA GGT GTT GGA TGC A-3'  (P) 5'-CTC TTC ATC TGT TTA GGG TCA TCA GCC TCA A-3' |
| IL-6 | (F) 5'-TTC CAA AAA TGG AGG AAA AGG A-3'  (R) 5'-TCC AGA AGA CCA GCA GTG GTT-3'  (P) 5'CTT CCA ATC TGG GTT CAA TCA GGC GAT T-3' |
| MMP1 | (F) 5'-TTC AGC TTT CTC AGG ACG ACA TT-3'  (R) 5'-CGA CTG GCT GAG TGG GAT TT-3'  (P) 5'-TCC AGG CCA TCT ACG GAC CTT CCC-3' |
| MMP3 | (F) 5'-GGC TGC AAG GGA CAA GGA A-3'  (R) 5'-CAA ACT GTT TCG TAT CCT TTG CAA-3'  (P) 5'-CAC CAT GGA GCT TGT TCA GCA ATA TCT AGA AAA C-3' |
| MMP9 | (F) 5'-ACG AAC CAA CCT CAC CAA CAG-3'  (R) 5'-TGC CCC AGG AGT GTA GCC-3'  (P) 5'-CAG CTG GCA GAG GAA TAC CTG TAC CGC-3' |
| MMP13 | (F) 5'-CCA TCT ACA CCT ACA CTG GCA AAA G-3'  (R) 5'-GTC TGG CGT TTT GGG ATG TT-3'  (P) 5'-TCT CTC TAT GGT CCA GGA GAT GAA GAC CCC-3' |
| TNF-α | (F) 5'-CCT CTT CTC AAG CCT CAA GTA ACA A-3'  (R) 5'-GAG CTG CCC CGG AGA GTT-3'  (P) 5'-ATG TCG GCT ACA ACG TGG GCT ACC G-3' |
| **Gene** | **Manufacturer, assay ID** |
| ELN | Life Technologies, Bt03216594_m1 |
| IL-8 | Life Technologies, Bt03211906_m1 |
| MMP19 | Life Technologies, Bt03248146_m1 |
| RPLP0 | Life Technologies, Bt03218086_m1 |
